# The impact of differences in plasma glucose between glucose oxidase and hexokinase methods on estimated gestational diabetes mellitus prevalence

**DOI:** 10.1038/s41598-019-43665-x

**Published:** 2019-05-10

**Authors:** Lynnsay M. Dickson, Eckhart J. Buchmann, Charl Janse Van Rensburg, Shane A. Norris

**Affiliations:** 1 MRC Developmental Pathways to Health Research Unit, Department of Paediatrics, Faculty of Health Sciences, University of the Witwatersrand, York Road, Parktown, Johannesburg South Africa; 2 Department of Obstetrics and Gynaecology, Faculty of Health Sciences, University of the Witwatersrand, York Road, Parktown, Johannesburg South Africa; 3 Biostatistics Unit, South African Medical Research Council, 1 Soutpansberg Road, Pretoria, South Africa

**Keywords:** Gestational diabetes, Translational research

## Abstract

We evaluated the extent of measurement discordance between glucose oxidase and hexokinase laboratory methods and the effect of this on estimated gestational diabetes mellitus (GDM) prevalence in a routine clinical setting. 592 consecutive urban African women were screened for GDM. Paired venous specimens were submitted to two independent calibrated laboratories that used either method to measure plasma glucose concentrations. World Health Organisation diagnostic criteria were applied. GDM prevalence determined by the glucose oxidase and hexokinase methods was 6.9% and 5.1% respectively. The overall GDM prevalence was 9%. Only 34% of GDM positive diagnoses were common to both laboratory methods. Bland Altman plots identified a bias of 0.2 mmol/l between laboratory methods. Plasma glucose concentrations measured by the glucose oxidase method were more platykurtic in distribution. Low diagnostic agreement between laboratory methods was further indicated by a Cohen’s kappa of 0.48 (p < 0.001). Reports of GDM prevalence using either the glucose oxidase or hexokinase laboratory methods may not be truly interchangeable or directly comparable.

## Introduction

Identifying usually asymptomatic gestational diabetes mellitus (GDM) will allow interventions to improve perinatal and long-term outcomes for both mother and her child^1,2^. In the Hyperglycaemia and Adverse Pregnancy Outcome study, which investigated the relationship between glycemia and birth outcomes, stringent research laboratory standards were applied across multiple research centres^3^. Evidence from controlled research laboratory settings, rather than routine clinical settings, informed the GDM clinical diagnostic criteria recommended by the International Association of Diabetes and Pregnancy Study Group (IADPSG)^1^. These recommendations were later adopted by the World Health Organisation (WHO)^2^. The WHO recommended gold standard 75 g oral glucose tolerance test (OGTT), which is used in both research and routine clinical settings to identify dysglycaemia, describes blood sample collection and transport methods^4^. However, it does not indicate a standardized laboratory method of measurement of plasma glucose concentrations^4^.

Laboratory methods commonly used to measure plasma glucose concentrations include the enzymatic glucose oxidase (GOx) and hexokinase (HK) methods^5^. It has been reported that results of the HK method are negatively biased relative to the GOx method^6,7^ but this does not affect the estimated prevalence of overt diabetes mellitus^5^. GDM has lower diagnostic thresholds than dysglycaemia in a non-pregnant adult^2,4^. Typically, GDM prevalence studies use either GOx^3,8–11^ or HK laboratory methods^12–14^ to measure plasma glucose concentrations. Meta-analyses on GDM prevalence often do not distinguish between laboratory methods used to measure plasma glucose concentrations^15–20^. The effect of measurement discordance between laboratory methods on the determined prevalence of GDM remains unknown. The presence of significant discordance between laboratory methods may not only limit the interpretation of global surveillance on the prevalence of GDM but will also impact the public health and clinical management of pregnant women.

We evaluated the extent of discordance between the GOx and HK laboratory methods on measured plasma glucose concentrations and the effect of this on the estimated prevalence of GDM. This pragmatic study was conducted in a public sector community health clinic using accredited laboratory services to mimic actual routine clinical conditions rather than a research setting. The primary outcomes of this data analysis were the clinical accuracy regarding a positive diagnosis of GDM, as well as the analytical accuracy regarding the level of agreement between paired laboratory results.

## Study Subjects, Methods and Materials

### Approvals and permissions

The study protocols were reviewed and approved by the University of the Witwatersrand Human Research Ethics committee (M150365). This study was conducted in accordance with the principles of the Declaration of Helsinki. The South African Johannesburg District Department of Health granted permission to conduct this study at a community health clinic (CHC) in Soweto, Johannesburg (2015–16/031). All participants provided written informed consent.

### Study design

This analysis is part of a study that investigated the utility of plasma-calibrated glucometers to universally screen for GDM in a low resource, routine clinical setting^21–23^. In this study both the HK and GOx laboratory methods were used as reference tests against the glucometers in all women tested. This prospective pragmatic cross-sectional study was conducted at a single CHC between April 2016 and May 2017. Consecutive women were recruited at their first antenatal clinic visit. Women aged 18 years and above and under 28 weeks of gestation were eligible for inclusion in the study. Women with type 1 or type 2 diabetes mellitus were excluded. All participants were urban African women, in keeping with this clinic’s patient profile.

### GDM screening procedures

Participants were recruited consecutively, and all completed an OGTT. In addition, all participants were evaluated for the presence of GDM risk factors which include maternal obesity (body mass index ≥30 kg/m^2^), maternal age ≥40 years, previous history of GDM, first degree relative with diabetes mellitus, previous unexplained intrauterine foetal death, previous macrosomic baby or complications in the current pregnancy which include polyhydramnios, foetus large for gestational age or the presence of repeated glycosuria^24^. A modified two-hour 75 g OGTT was scheduled at the woman’s convenience on a morning after an overnight fast at 24–28 weeks’ gestation^2^. OGTT modifications included the collection of two venous blood samples at fasting, 60 and 120 minutes per participant. Venous blood samples were collected in tubes containing the glycolytic inhibitor sodium fluoride^4^ (BD 454297) and kept on ice from the time of phlebotomy until delivery to the laboratories within an hour of completion of the OGTT. The immediate separation of plasma or keeping samples on ice and separating plasma within 30 minutes was not feasible. Although these separation procedures are recommended by the American National Academy of Clinical Biochemistry (NACB), they and other researchers acknowledge that these procedures are uncommon in routine clinical settings^5,25–27^. Trained research staff operated independently from the CHC staff.

### Laboratory services

There is no laboratory on-site at CHCs in South Africa. The laboratories used in this study were 14 km (research medical laboratory) and 16 km (private medical laboratory) distant. A South African National Accreditation System verified private medical laboratory used the HK method (Cobas 6000, Roche Diagnostics International, Switzerland) and a medical research laboratory used the GOx method (Randox, Daytona Plus, USA) exclusively to determine plasma glucose concentrations. Study samples were processed within the usual functioning of these laboratories. Laboratories were not requested to adjust their routine standard operating procedures for this study. On request, laboratories provided us with their quality control results, namely, the analytic coefficient of variation (CV) and bias at two glucose quality control levels. For the HK method laboratory, at observed mean glucose concentrations of 3.32 mmol/l and 20.16 mmol/l, the CV was 1.79% and 1.74% and the bias was 0.36% and 0.13% respectively. For the GOx method laboratory, at observed mean glucose concentrations of 6.36 mmol/l and 15.80 mmol/l, the CV was 1.85% and 1.67% with a bias of 0.35% and 0.31% respectively. The NACB recommendation for total maximal allowable error in laboratory measurement of plasma glucose is ≤6.9% with an imprecision (CV) of ≤2.9% and a bias of ≤2.2%^5^.

### Sample handling

In this study, all venous blood samples were subject to similar preanalytical conditions. Paired venous blood samples were collected from a single venous draw, meaning that intra-individual biologic factors were unlikely to affect interlaboratory method comparisons^28^. It is known that blood glucose concentration drops by approximately 5–7% per hour *ex-vivo* due to ongoing glycolysis, and sodium fluoride does not necessarily negate this effect within the first four hours^5,28^. The use of sodium fluoride tubes is included in the WHO recommended OGTT procedure^4^. All samples were subject to similar transport conditions and ongoing glycolysis was unlikely to affect interlaboratory method comparisons. All venous blood samples were delivered to the off-site laboratories within an hour of completion of the OGTTs and within 15 minutes of each other. Meaning the blood samples for fasting, 60 and 120 minutes reached the laboratory after three, two and one-hour post phlebotomy respectively. Each laboratory processed samples within one hour of receipt and this includes centrifugation and measurement of plasma glucose concentration. For reasons of convenience, samples were delivered to the GOx laboratory before the HK laboratory. This study was conducted over a period of 14 months and so results were unlikely to be affected by a specific laboratory analytical run. The two laboratories operated independently and were blinded to all but their own results.

### Clinical diagnostic criteria

The WHO 2013 GDM clinical diagnostic criteria were used to define test positivity cut-offs for results from each laboratory method^2^.

### Statistical analysis

Categorical variables are described as frequencies (n) and proportions (%) and continuous variables as means and standard deviations (SD). The Bland Altman method was used to assess the level of agreement between HK and GOx methods and results are shown with 95% limits of agreement (95% LoA). McNemar’s test, the kappa statistic, and Lin’s concordance correlation coefficient were also used to evaluate the agreement between paired plasma glucose results. The kappa-statistic (κ) values were graded as <0.20 = poor, 0.20–0.39 = fair, 0.40–0.59 = moderate, 0.60–0.79 = good and ≥0.80 = very good, regarding levels of agreement. The paired t-test was used to assess if the mean difference in glucose results between methods were different from zero. A p value of <0.05 was considered to indicate statistical significance. Statistical analysis was performed using STATA software version 15 (Stata Statistical Software: Release 15. College Station, TX: StataCorp LLC, USA).

## Results

### Participant characteristics

One or more risk factors for GDM was present in 257 (43.4%) of 592 participants. Clinical characteristics strongly associated with a GDM positive diagnosis include increased maternal age (p < 0.001), an increased body mass index (p = 0.001) and a later presentation for their first antenatal clinic visit (p = 0.001) (Table 1). Overall, participants were overweight with a mean body mass index (BMI) of 26.9 kg/m^2^. In addition, 173 (29.2%) of 592 participants were pregnant for the first time.

**Table 1:** Participant Clinical Characteristics.

Clinical characteristic	All participants		Composite laboratory GDM Positive		p-value
	Number of participants	Value N (%) or Mean (SD)	Number of participants	Value N (%) or Mean (SD)	
Age, (Years)	592	27.8 (5.9)	53	31.4 (6.8)	<0.001
Family history of diabetes	588	99 (16.8)	52	13 (25.0)	0.099
Glycosuria (urine dipstick)	592	6 (1.0)	53	4 (7.6)	—
Mid upper arm circumference (cm)	592	29.9 (4.2)	53	31.9 (4.3)	<0.001
Body height (cm)	588	162.1 (6.6)	52	160.7 (6.6)	0.103
Body weight (Kg)	592	70.6 (15.8)	53	76.8 (15.1)	0.003
BMI (Kg/m²)	588	26.9 (5.8)	52	29.5 (5.6)	0.001
Obstetric Characteristics			53		
Gestational at first visit (weeks)	592	19.1 (5.6)	53	20.8 (5.7)	0.001
Number of pregnancies including current 1 2 ≥3	592	173 (29.2) 196 (33.1) 223 (37.6)		7 (13.2) 12 (22.6) 34 (64.2)	0.007 (1 vs 2+) <0.001 (≤2 vs 3+) 0.415 (≤3 vs 4+)
Previous large for gestational age birth	591	43 (7.3)	53	8 (15.1)	0.045
Previous stillbirth	592	32 (5.4)	53	3 (5.7)	1.000
Previous congenital abnormalities	591	0 (0)	53	0 (0)	—
Previous GDM	592	3 (0.5)	53	1 (1.9)	—

Note: Number of participants for each characteristic varies slightly due to missing values.

### Availability of results

The private laboratory provided results within four hours of receiving samples and the research laboratory provided results at the end of the week of testing. Participants were informed of their GDM status and those identified as being GDM positive, by either laboratory method, were referred for clinical intervention. Complete OGTTs for 592 women, in total 1776 paired plasma glucose results, were submitted for analysis. The mean (SD) glucose concentrations at fasting, 60 and 120 minutes of the OGTT for the GOx method were 4.01 mmol/l (3.95–4.07 mmol/l), 5.42 mmol/l (5.30–5.54 mmol/l) and 5.12 mmol/l (5.01–5.23 mmol/l) respectively. For the HK method, these were 4.13 mmol/l (4.09–4.17 mmol/l), 5.62 mmol/l (5.51–5.73 mmol/l) and 5.27 mmol/l (5.17–5.37 mmol/l) respectively. For all 1776 observations, the interquartile range for the GOx and HK methods was 1.70 mmol/l and 1.55 mmol/l respectively.

### Clinical accuracy

**Figure 1:**
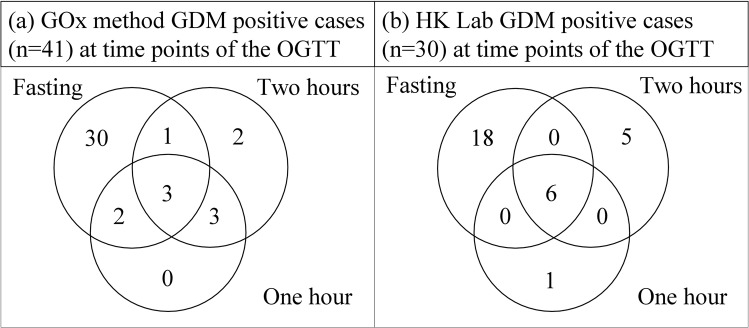
Venn diagrams illustrating the number of GDM positive cases identified at various time points of the OGTT by the GOx (Glucose Oxidase) and HK (Hexokinase) laboratory methods. (WHO 2013 test positivity cut-offs).

The prevalence (95% Confidence Interval) of GDM as defined by the GOx method was 6.9% (5.0–9.3%) and for the HK method, 5.1% (3.4–7.2%). The composite laboratory GDM prevalence was 9.0%, meaning that 53 of 592 participants were GDM positive as defined by either or both laboratories. Regarding clinical agreement, 18 of the total 53 GDM positive diagnoses (34%) were common to both laboratory methods. The GOx method identified 23 additional participants as having GDM that were not identified by the HK method. The HK method identified 12 additional positive GDM cases not identified by the GOx method. An elevated fasting plasma glucose was diagnostic in 87.8% of GOx method and 80.0% of HK method GDM positive cases respectively. GDM positive diagnoses based on abnormalities at various time points of the OGTT as determined by the GOx and HK methods are displayed as Venn diagrams in Fig. 1. The overall observed clinical agreement in GDM status between the GOx and HK laboratory methods was high (94.1%) because of the high proportion of GDM negative cases. The overall Cohen’s kappa statistic was calculated as 0.48 (p < 0.001), which suggests a moderate agreement between the two laboratory methods in terms of clinical accuracy in diagnosing GDM. Using a significance level of 5%, McNemar’s test indicates no significant disagreement in GDM diagnoses between laboratory methods (χ^2^ = 3.46; p = 0.0895).

### Laboratory analytical accuracy

**Figure 2:**
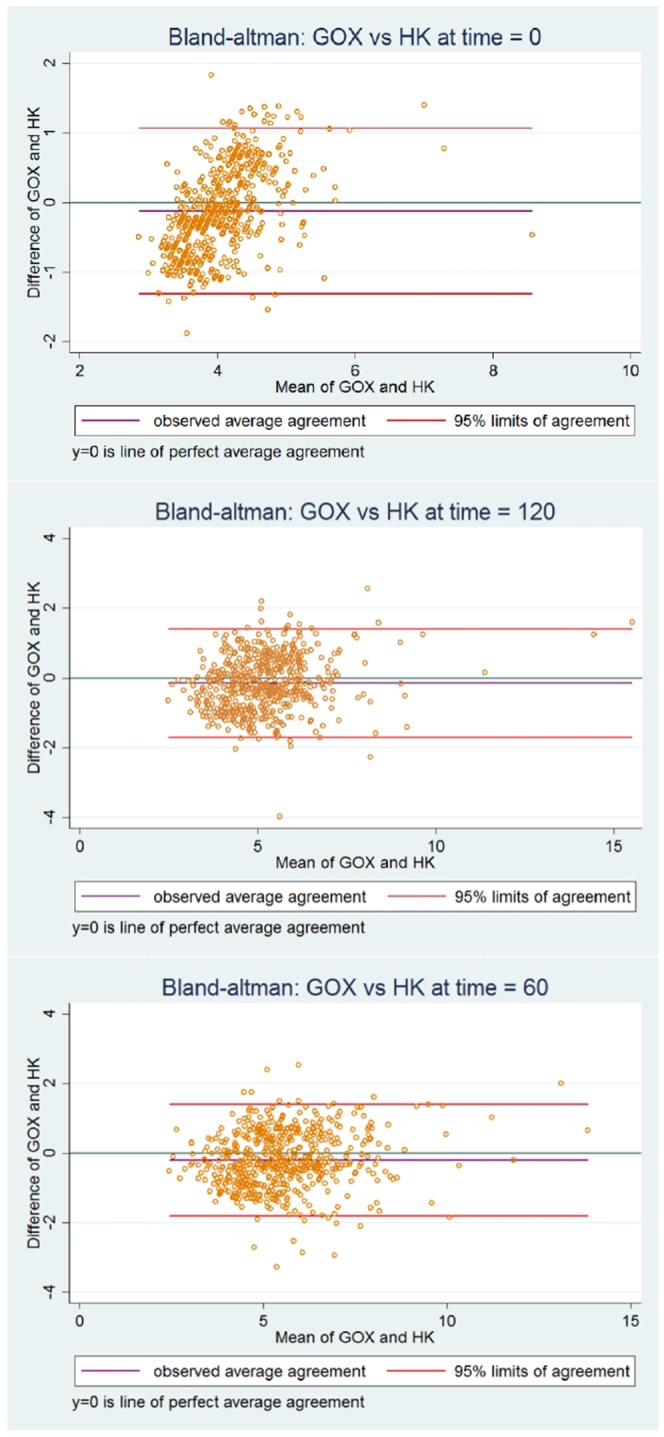
Bland Altman plots of the paired plasma glucose results (mmol/l) measured by the GOX (Glucose Oxidase) and HK (Hexokinase) methods at 120 minutes of the 75 g oral glucose tolerance tests for 592 women screened for gestational diabetes.

Of the paired laboratory results at fasting, 60 and 120 minutes of the OGTT, the average difference against the mean (95% LoA) were −0.12 mmol/l (−1.31–1.07 mmol/l), −0.20 mmol/l (−1.80–1.39 mmol/l) and −0.15 mmol/l (−1.70–1.40 mmol/l) respectively. On average, the HK laboratory method results were positively biased (higher) relative to the GOx method results at all time points of the OGTT. Bland Altman plots for paired results for each time points of the OGTT are presented in Fig. 2. The overall average difference against the mean between the two methods for all 1776 observations was −0.20 mmol/l (−1.6–1.3 mmol/l). The systematic bias between methods is indicated by the 95% Confidence Interval for the mean difference (−0.2 to −0.1 mmol/l), which does not cross a zero baseline. This is confirmed by the mean difference between methods being statistically different from zero at each time point of the OGTT (p < 0.001). The concordance correlation coefficients (95% Confidence Interval) between the GOx and HK methods at fasting, 60 and 120 minutes of the OGTT were 0.53 (0.48–0.58), 0.83 (0.81–0.86) and 0.81 (0.78–0.84) respectively.

## Discussion

Although each laboratory fulfilled the NACB recommended analytic accuracy standard, there was a low agreement regarding GDM positive status. Possible reasons for this clinical difference are the systematic bias of 0.20 mmol/l revealed by the Bland Altman analysis as well as the difference in the distribution of plasma glucose results, the kurtosis, between methods. The mean plasma glucose concentration measured by the HK method was higher than that of the GOx method at each time point of the OGTT, but the HK method identified a lower prevalence of GDM. The interquartile range of measures from the HK method was relatively lower by 0.15 mmol/l and this suggests that the HK method produced a narrower distribution of measures contained within the distribution of measures from the GOx method. This, in turn, indicates that measures from the GOx method are both lower in the lower range and higher in the higher range relative to the HK method. The HK method is known to be more specific than the GOx method^29^. Substances such as uric acid, ascorbic acid, bilirubin, hemoglobin, tetracycline, and glutathione may cause falsely low glucose results measured by the glucose oxidase method^29^.

Internationally, inconsistent laboratory methods are used to measure plasma glucose concentrations and there is no recognized gold standard reference test^30^. The American Centre for Disease Control has previously suggested isotope dilution gas chromatography-mass spectrometry as the gold standard reference test, but this was not adopted by the NACB^5,30^. Laboratories measure glucose almost exclusively by the enzymatic HK or GOx methods with few laboratories using the glucose dehydrogenase method^5^. However, the IADPSG recommendations, which were largely based on the clinical outcomes results of the HAPO study, are based on plasma glucose concentrations measured by the GOx method^3^. In South Africa, the HK laboratory method is used in the state-sponsored National Health Laboratory Service as well as the two largest private medical laboratories. It is possible that the HK method may underestimate the prevalence of GDM and fail to identify women at high risk of poor perinatal outcomes as identified in the HAPO study population.

This pragmatic study was conducted in a low resource urban African CHC which had the advantage of access to off-site accredited laboratory services. The availability of these services should not be assumed as it is known that Sub-Saharan Africa has minimal or no access to internationally accredited medical laboratory services^31^. This translational research study, applied in a routine clinical setting, serves to raise awareness of discordance between laboratory methods as a further limitation to the global comparison on GDM prevalence estimates. The analytic accuracy of routine but varying laboratory methods of measuring glucose may be unable to support the clinical accuracy required by the WHO GDM evidence-based diagnostic guidelines. Implementation of universal screening programs for GDM with medical decisions based on poorly reproducible laboratory results may adversely affect both the women affected and the optimal utilization of public health systems resources.

### Limitations

The low prevalence of GDM and the small proportion of GDM positive diagnoses common to both methods, limits the precision of the reported estimates. The potential clinical diagnostic uncertainty between laboratory methods should be explored in larger groups of patients and in more diverse clinical settings.

## Data Availability

Data will be made available on request.
